# Effects of exogenous sulfur on alleviating cadmium stress in tartary buckwheat

**DOI:** 10.1038/s41598-019-43901-4

**Published:** 2019-05-14

**Authors:** Yang Lu, Qi-fu Wang, Jun Li, Jiang Xiong, Luo-na Zhou, Sheng-ling He, Jie-qiong Zhang, Zhong-ai Chen, Song-gang He, Hui Liu

**Affiliations:** 1 College of Food and Pharmaceutical Engineering, Guizhou Institute of Technology, Guiyang, 550003 China; 2 Guizhou Biotechnology Institute, Guizhou Academy of Agricultural Sciences, Guiyang, Guizhou 550006 China; 3 Guizhou Key Laboratory of Agricultural Biotechnology, Guiyang, Guizhou 550006 China; 4 Guizhou general station of crop technology promotion, Guiyang, Guizhou 550001 China

**Keywords:** Plant physiology, Cell wall, Fertilization

## Abstract

Supplying exogenous sulfur-rich compounds increases the content of glutathione(GSH) and phytochelatins(PCs) in plant tissues, enabling plants to enhance their cellular defense capacity and/or compartmentalize Cadmium(Cd) into vacuoles. However, the mechanism by which surplus S modulates tolerance to Cd stress in different tissues need further investigation. In the present study, we found that supplementing the tartary buckwheat(*Fagopyrum tararicum*) exposed to Cd with surplus S reversed Cd induced adverse effects, and increased Cd concentrations in roots, but decreased in leaves. Further analysis revealed that exogenous S significantly mitigated Cd-induced oxidative stress with the aids of antioxidant enzymes and agents both in leaves and roots, including peroxidase(POD), ascorbate peroxidase(APX), glutathione peroxidase(GPX), glutathione S-transferase(GST), ascorbic acid(AsA), and GSH, but not superoxide dismutase(SOD) and catalase(CAT). The increased Cd uptake in root vacuoles and decreased translocation in leaves of exogenous S treated plants could be ascribed to the increasing Cd binding on cell walls, chelation and vacuolar sequestration with helps of non-protein thiols(NPT), PCs and heavy metal ATPase 3(FtHMA3) in roots, and inhibiting expression of *FtHMA2*, a transporter that helps Cd translocation from roots to shoots. Results provide the fundamental information for the application of exogenous S in reversal of heavy metal stress.

## Introduction

Cadmium (Cd) has become a primary heavy metal pollutant due to its increasing concentration in agricultural farmland, which, in turn, reduces crop production and threatens human health as it easily enters the food chain^1,2^. Cd alters the chloroplast structure, disrupts the photosynthetic process, and increases reactive oxygen species (ROS), such as O_2_^−1^, ·OH, and H_2_O_2_^3^. Consequently, excessive ROS results in lipid peroxidation, protein oxidation, nucleotide (DNA and RNA) damage, and ultimately inhibits the growth and productivity of crops^4^.

A series of mechanisms have been developed by plants to counteract Cd-induced oxidative stress, including enzymatic and non-enzymatic antioxidative reactions and the production of thiol compounds^5,6^. Several strategies have been applied to neutralize Cd-induced toxicity in plants, and supplementing with mineral elements is the best strategy. Supplying sulfur (S) results in enhanced photosynthesis via upregulated expression of stromal and thylakoid proteins, and strengthening of stomatal movement under Cd stress^6–8^.

S is an essential nutrient element for cysteine (Cys), methionine, reduced glutathione (GSH), coenzyme A, sulfo-lipids, iron-sulfur (Fe-S) clusters, and the thioredoxin system, and plays a vital role in the stress tolerance of plants^9^. GSH acts as a non-enzymatic antioxidant that scavenges excessive ROS and balances redox homeostasis through the ascorbate(AsA)-GSH cycle^4,10^. S assimilation is upregulated in plants under various biotic and abiotic stressors^11^. The Cd-mediated induction of S-metabolite production is associated with a mitigation of Cd cytotoxicity^12^. Consequently, supplementing with S is an important approach to alleviate Cd stress in plants^13,14^. However, a growing number of studies indicate that surplus S is a two-edged sword in crops. Low S content facilitates crop growth, while high S content restrains nitrogen uptake, which reduces crop production^15^. Therefore, an appropriate level of S is of central importance to reverse Cd cytotoxicity and maintain the regular growth of crops.

Tartary buckwheat (*Fagopyrum tararicum*) is an edible, medicinal crop that can be cultivated on infertile and frigid farmlands. Previous reports show that tartary buckwheat is highly aluminum (Al) tolerant because it secretes oxalate from the roots^16^. Preliminary experiments indicate that tartary buckwheat also tolerates high concentrations of Cd (>200 mg·L^−1^ of CdCl_2_), and that exogenous S supplied at an appropriate concentration of 400 mg·L^−1^ plays an important role alleviating Cd stress. Previous studies have revealed the essential role of S in the biosynthesis of GSH, glutathione S-transferase (GST), and phytochelatins (PCs), i.e, which scavenge various ROS and/or chelate Cd to reduce the deleterious effects of Cd on plants, and decrease Cd transportation from roots to shoots^17–20^. Heavy metal ATPase 3 (HMA3), a P_1B2_-ATPase involved in direct compartmentalization of Cd into vacuoles in rice, is significantly more highly expressed in Cd-exposed rice roots than in control plants, and reduces Cd content in leaves and grains^21^. Additionally, overexpression of the plasma membrane Cd extruder *HMA2* in roots, stems, and leaves of *Sedum alfredii* results in higher Cd content in leaves, indicating a vital function of HMA2 for efficient Cd translocation from roots to shoots^22^. However, the effect of surplus S on the expression levels of these genes and their connection to Cd translocation in tartary buckwheat has not been investigated. In addition, whether there are different mechanisms in leaves and roots for Cd accumulation and detoxification in tartary buckwheat remain unclear. Therefore, in the present study, we evaluated the effects of supplementary S on Cd tolerance and distribution in tartary buckwheat. We also investigated the interaction between S supply and the expression of genes involved in Cd vacuolar sequestration and transportation in tartary buckwheat seedlings. The aim of the current study was to uncover the physiological and molecular mechanism associated with the effect of exogenous S on Cd accumulation and distribution in tartary buckwheat.

## Materials and Methods

### Plant materials and experimental design

Tartary buckwheat (*F. tararicum* Gaertn. cv. Qianku 4) seeds of a uniform size were surface sterilized in 0.1% mercuric chloride solution for about 10 min, rinsed three times in distilled water, and then placed on wet filter paper in Petri dishes to germinate in the dark at 25 ± 1 °C. About 72 h later, five healthy seedlings of equal size were selected and placed on a net tray floating on 1/2 strength Hoagland solution (pH 6.0) in a 2-L square plastic container. The Hoagland solution was prepared according to Hoagland and Arnon^23^ with slight modifications to contain 506 mg·L^−1^ KNO_3_, 945 mg·L^−1^ Ca(NO_3_)_2_, 80 mg·L^−1^ NH_4_NO_3_, 136 mg·L^−1^ KH_2_PO_4_, 493 mg·L^−1^ MgSO_4_, 2.5 mL ferric salt solution (5.56 g·L^−1^ FeSO_4_·7H_2_O, 7.46 g·L^−1^ Na_2_-EDTA, pH = 5.5), 0.83 mg·L^−1^ KI, 6.2 mg·L^−1^ H_3_BO_4_, 22.3 mg·L^−1^ MnSO_4_, 8.6 mg·L^−1^ ZnSO_4_, 0.025 mg·L^−1^ CuSO_4_, 0.025 mg·L^−1^ CoCl_2_, and 0.25 mg·L^−1^ Na_2_MoO_4_, which were renewed every 2 days. Tartary buckwheat plants were grown in a culture room with a 14-h photoperiod, and average day/night temperatures of 25 ± 1 °C and 20 ± 1 °C, respectively. Relative humidity was maintained at 75%. Each treatment was repeated at least three times.

The optimal treatment concentrations of Cd^2+^ (CdCl_2_·2.5 H_2_O; Sigma, St. Louis, MO, USA) and SO_4_^2^^−^[(NH_4_)_2_ SO_4_, Sigma)] were obtained via preliminary experiments, and were 100 mg·L^−1^ and 400 mg·L^−1^, respectively. Plants supplied only with the nutrient solution served as controls. The 100 mg·L^−1^ CdCl_2_·2.5 H_2_O and/or 400 mg·L^−1^ (NH_4_)_2_SO_4_ treatments were applied 10 days after sowing (DAS), and additional NH_4_Cl was added to the treatments without (NH_4_)_2_SO_4_ to maintain a uniform NH_4_^+^ concentration. At 20 DAS, the top second leaves and roots were collected to determine various parameters. Plant height and root length were measured using a ruler. The dry weights of root and shoot samples were determined after oven drying at 110 °C for 1 h and then at 70 °C to constant weight. All parameter measurements were repeated three times.

### Measurement of photosynthesis

Net photosynthesis was estimated in the fully expanded uppermost leaves of the plants using LI-6400 photosynthesis determination system (LI-COR Inc., Lincoln, NE, USA). The measurements were conducted between 10 a.m. and 2 p.m. on a sunny day. The parameters were set as follows: light intensity was 1,400 μmol·m^−2^·s^−1^, and atmospheric CO_2_ concentration was 380 ± 5 mmol·mol^−1^.

### Estimates of superoxide anion (O_2_^−^) and H_2_O_2_ contents

The superoxide anion (O_2_^−^) content in leaves and roots of tartary buckwheat were determined according to the protocol described by Adhikari *et al*.^20^ with slight modifications. About 0.1 g of freshly collected leaves (pretreated with light of 1,000 μmol·m^−2^·s^−1^) and roots were cut into small segments (100 mg) and immersed in 2 mL of reaction buffer [20 mM Na_2_-EDTA, 40 μM NADH and 50 mM phosphate buffer (pH 7.8)]. The reaction was initiated by supplementing 100 μL of 25 mM epinephrine (newly prepared in 0.1 M HCl), and the mixtures were incubated at 28 °C for 15 min on a rotary shaker with a rotating speed of 150 rpm. Thereafter, the absorbance of the reaction mixtures was detected at 480 nm using an ultraviolet spectrophotometer (UV-2600, Shimadzu Ltd., Tokyo, Japan) after the tissues were thoroughly removed. O_2_^−^ content was calculated by the rate of adrenochrome production and represented as nmol·g^−1^ FW.

The hydrogen peroxide (H_2_O_2_) content in leaves and roots of tartary buckwheat were determined according to the method of Khan *et al*.^14^. About 0.5 g of fresh leaf or root tissues were ground in 10 mL pre-chilled 200 mM perchloric acid. The homogenates were centrifuged at 1,200 × g and 4 °C for 10 min. After measuring the volume of the supernatant, the supernatant was added to another centrifugation tube and mixed with isopyknic 4 M KOH. After centrifugation at 500 × g and 4 °C for 5 min, the insoluble precipitate was discarded. Two mL of the supernatant was mixed with 800 µL of 12.5 mM 3-(dimethylamino) benzoic acid in 0.375 M phosphate buffer (pH 6.5), 160 µL of 3-methyl-2-benzothiazoline hydrazone, and 40 µL of peroxidase (0.25 unit). The increase in absorbance was read at 590 nm using the ultraviolet spectrophotometer.

### Measurement of malondialdehyde (MDA) content and relative electrolytic leakage (REL)

The MDA content was determined following the protocol reported by Hui *et al*.^24^ with minor modifications. About 0.5 g of leaves or roots were ground with a mortar and pestle in 10 mL of 10% (w/v) trichloroacetic acid (TCA) and then centrifuged at 4,000 × g for 10 min. A mixture of 2 mL of the supernatant and 2 mL of 0.7% (w/v) 2-thiobarbituric acid was boiled at 100 °C for 15 min. After quickly cooling on ice, the mixture was centrifuged at 4,000 × g for 10 min, and the absorbance of the supernatant was recorded at 450, 532, and 600 nm with an ultraviolet spectrophotometer (UV-2700, Shimadzu).

To measure REL, 0.5 g fresh tissue was immersed in 30 mL deionized water (dH_2_O). After continuous shaking for 24 h at room temperature, the electrolyte content in the dH_2_O was tested immediately (C0) with a conductivity meter (DDSJ-318, Leici, Shanghai, China). The electrolyte content in the solution was determined (C1) by boiling for 20 min. REL was calculated according to the following equation: REL = (C0/C1) × 100%^24^.

### Determination of antioxidant enzyme activities

A 0.5 g aliquot of fresh leaf or root was homogenized in 6.0 mL 0.05 M sodium phosphate buffer [PBS buffer containing 1% (w/v) polyvenylpyrrolidone (PVP), 1% (v/v) Triton-X 100, 100 mM EDTA, pH 7.8] on ice using a pre-chilled (4 °C) mortar and pestle, and then stewed for 10 min on ice. The homogenate was centrifuged at 12,000 × g for 20 min at 4 °C. Superoxide dismutase (SOD) and catalase (CAT) activities were assayed in the supernatant according to previously described methods (Rama and Prasad 1998)^25^.

About 0.5 g of frozen leaf or root were ground in 5 mL pre-chilled 0.5 M phosphate buffer (containing 1% (w/v) PVP, 1% (v/v) Triton-X 100, 100 mM EDTA, pH 7.8) using a cold pestle and mortar on ice. The homogenates were centrifuged at 8,000 × g for 15 min at 4 °C. The ascorbate peroxidase (APX) activity and GST activity in the supernatant were measured according to the methods of Bashir *et al*.^13^ and Liang *et al*.^18^, respectively. The reaction mixture contained 50 mM PBS buffer (pH 7.0), 0.5 mM ascorbate (AsA), 2 mM H_2_O_2_, and 300 µL supernatant including the APX enzyme in a final volume of 3 mL to detect APX activity. The reaction mixture for the GST assay contained 0.1 M PBS buffer (pH 6.5), 1 mM EDTA, 1 mM 1-chloro-2,4-dinitrobenzene (CDNB), 1 mM GSH, and 300 µL of the enzyme extract. All reaction reagents were freshly prepared before the test started. The reaction mixture was equilibrated at 25 °C for 1 min before initiating the reaction by adding H_2_O_2_ or CDNB. The reduction in absorbance at 290 nm and the increase in absorbance at 340 nm were recorded during 3 min to determine APX and GST activities, respectively. The reaction buffer without AsA or GSH was used as the control. A unit of APX and/or GST was defined as a 0.01 decrease/increase in absorbance at 290/340 nm for 1 min at 25 °C and expressed as U·mg^−1^ FW.

Peroxidase (POD) activity was detected by using a Plant Peroxidase assay kit (Fusheng Ltd., Shanghai, China). Glutathione peroxidase (GPX) was determined according to the protocol contained in the Glutathione Peroxidase assay kit (BioVision Inc., San Francisco, CA, USA).

### Enzyme activity assays for the AsA–GSH cycle, AsA and GSH (reduced) contents, ratio of AsA/DHA, and the redox state

To prepare the crude enzymes extracts, 0.5 g of leaf or root tissues were ground into a powder in liquid nitrogen using a pre-chilled mortar and pestle. The powder was homogenized in 10 mL of pre-cooled 50 mM PBS buffer (pH 7.0) containing 1.0 mM EDTA, 0.05% (v/v) Triton X-100, 2% (w/v) PVPP, 1 mM AsA, and 0.5 mM phenylmethylsulfonyl fluoride on ice. The homogenates were centrifuged at 16,000 × g for 15 min at 4 °C. The supernatants were collected and applied to detect enzyme activities. The activities of glutathione reductase (GR) and monodehydroascorbate reductase were determined following the methods of Liang *et al*.^18^.

A 10 g portion of fresh leaves or roots was homogenized in 5.0 mL 2% (w/v) oxalate solution on ice using a pre-chilled (4 °C) mortar and pestle to measure AsA content. The homogenate was transferred to a 100 mL volumetric flask, and 2% (w/v) oxalate solution was added to the flask until the metered volume was reached. After homogenization via shaking, the homogenate was filtered using four layers of gauze and subsequently centrifuged at 10,000 × g for 30 min at 4 °C. The supernatant was used to determine AsA content. The 2, 6-dichlorophenol-indophenol-based titration method was applied to determine AsA content according to the protocol described by Liang *et al*.^18^. Total AsA [AsA + dehydroascorbate (DHA)] was assayed after reducing DHA to AsA using dithiothreitol (DTT), and the difference between total AsA and AsA was determined as DHA content.

A 2.5 g portion of fresh leaves or roots was homogenized in 5 mL of pre-chilled 5% (w/v) TCA (containing 5 mM Na_2_-EDTA) on ice using a pre-chilled (4 °C) mortar and pestle. The homogenate was centrifuged at 12,000 × g for 20 min at 4 °C. The supernatant was used for total GSH [GSH + glutathione disulfide (GSSG)], and GSSG content was determined according to the method described by Khan *et al*.^8^ GSH content was calculated from the difference between total GSH and GSSG.

### Determination of ATP sulfurylase activity and total S, NPT, and PC contents

ATP-sulfurylase (ATP-S) activity was determined according to the protocol described by Khan *et al*.^14^. About 1.0 g of fresh tissue (leaf or root) was ground using a mortar and pestle at 4 °C in extraction buffer containing 10 mM Na_2_-EDTA, 20 mM Tris-HCl (pH 8.0), 2 mM DTT, and 0.01 g·mL^−1^ PVP, with a tissue: buffer ratio of 1:4 (w/v). After centrifugation at 20,000 × g for 10 min at 4 °C, the supernatant was applied to estimate ATP-S activity. In the final volume of 3 mL, the reaction mixture contained 7 mM MgCl_2_, 5 mM Na_2_MoO_4_, 2 mM Na_2_ATP, and 0.032 units·mL^−1^ of sulfate-free inorganic pyrophosphate in 80 mM Tris-HCl (pH 8.0). The reaction was initiated by adding 600 μL of the extract to the reaction mixture. Another aliquot from the same extract was added to the same reaction mixture but without Na_2_MoO_4_. The mixtures were incubated at 37 °C for 15 min, and phosphate was determined.

Total sulfur content was measured in leaf and root samples digested in HNO_3_: HClO_4_ (4:1, v:v) by boiling at 160 °C to near dryness. Thereafter, an atomic absorption spectrophotometer (iCE 3300 AAS, Thermo Scientific, Waltham, MA, USA) was used to detect total S content.

NPTs were extracted by homogenizing plant tissues (1 g) in 10% (w/v) sulfosalicylic acid solution. The homogenate was centrifuged for 8 min at 12,000 × g and 4 °C, and the supernatants were subsequently applied to detect sulfhydryl groups and GSH. NPT content was estimated spectrophotometrically with Ellman’s reagent according to Sedlak and Lindsay^26^: 250 µL of the supernatant was mixed with 500 µL of PBS (0.2 M, pH 7.5) and 20 µL of 0.6 mM 5,5′dithiobis[2-nitrobenzoic acid] (DTNB), and absorbance was measured 412 nm. Total glutathione was estimated in accordance with the protocol described by Anderson (1985)^27^. The PC contents in plant tissues were calculated by subtracting the total GSH content from the total amount of NPT^28^.

### Determination of Cd concentration

Plant tissues (leaf and root) were rinsed three times with dH_2_O, and were then separated into two parts. One part was used to determine total Cd, which was first dried at 110 °C for 30 min, and then at 70 °C for 24 h. The dried tissues were digested in HNO_3_: HClO_4_ (4:1, v:v) by boiling at 160 °C to near dryness. The other part was used to estimate subcellular Cd distribution, which was implemented by following the method reported by Hui *et al*.^24^ with a slight modification: 0.5 g fresh leaf or root was homogenized in 10.0 mL prechilled extraction buffer [250 mmol·L^−1^ sucrose, 50 mmol·L^−1^ Tris-HCl (pH 7.5), 10 mmol·L^−1^ DTT] on ice using a pre-chilled (4 °C) mortar and pestle. The homogenates were centrifuged for 10 min at 300 rpm. The precipitates were the cell wall fractions. The supernatants were subjected to another centrifugation at 2,000 rpm for 15 min, and then at 10,000 rpm for 20 min. The precipitate was the organelle fraction containing mitochondria and/or chloroplasts, and the supernatant was the soluble fraction consisting of vacuoles.

The Cd concentration was estimated in all fractions using an atomic absorption spectrophotometer (iCE 3300 AAS, Thermo Scientific).

### *FtHMA3* and *FtHMA2* expression analysis

Total RNA was isolated with the Trizol kit (Takara, Shiga, Japan) according to the manufacturer’s directions. A 2 µg portion of total RNA served as the template to synthesize the first-strand cDNA using the oligo (dT) primer and Prime-ScriptRTase (Takara). For quantitative real-time RT-PCR (qRT-PCR), a StepOnePlus Real-Time PCR system (ABI, Foster City, CA, USA) was applied to quantify the expression levels of *FtHMA3* and *FtHMA2* using SYBR Premix Ex Taq II (Takara) in a final reaction volume of 25 μl (containing 0.4 mM of each primer, and 2 µg of cDNA template). The primers employed for *FtHMA3* were designed based on the gene sequences of *BdHMA3* (*Brachypodium distachyon*, XM_003561234), *OsHMA3* (*Oryza sativa*, XM_015791882), and *ZmHMA3* (*Zea mays*, XM_020548450.2), which were F (5′-GGTTACATTGCCGTGAGGACGAC-3′) and R (5′-TCGACAAGACCGGCACCATCACC-3′). The *FtHMA2* primers were designed based on the gene sequence of *ZmHMA2* (*Z. mays*, NM_001360049.1), and the primer sequences were as follows: F: (5′-GAGCCGAGATGGCGCTGCTCG-3′), R: (5′-GTCAAGCCGTGCAGCTGATCG-3′). The actin gene with the F (5′-TCGAGACTGCGAAGAGTAGC-3′) and R (5′-TCCATGCCGATGATGGAAGG-3′) primers and the histone H3 gene with the F (5′- GGTCAACTTGTTGATTCCCCTCT-3′) and R (5′-AACCGCAAAATCCAAAGAACG-3′) primers were used as reference genes for qRT-PCR. The primer sequences were designed with Primer 5.0 software. The qRT-PCR cycling conditions were as follows: 95 °C for 10 min, followed by 35 cycles of 95 °C for 30 s, 59 °C for 30 s, and 72 °C for 30 s. The 2^−*ΔΔCt*^ method^29^ was applied to normalize the gene expression, and the relative expression level of *FtHMA3* was calculated. Each experiment was repeated three times.

### Statistical analysis

SPSS v18.0 statistical software (SPSS Inc., Chicago, IL, USA) was used for statistical analyses. Sample variability of each index was expressed as the standard error (SE). All data are expressed as mean ± SE (n = 3, *P* < 0.05). All data sets were checked for normality and heteroscedasticity using Levene’s test. Thereafter, one-way analysis of variance and Duncan’s test were applied to estimate the significance of the effects among the various treatments. Differences were considered significant at *P* < 0.05.

## Results

### Exogenous sulfur protects tartary buckwheat from Cd toxicity

**Figure 1:**
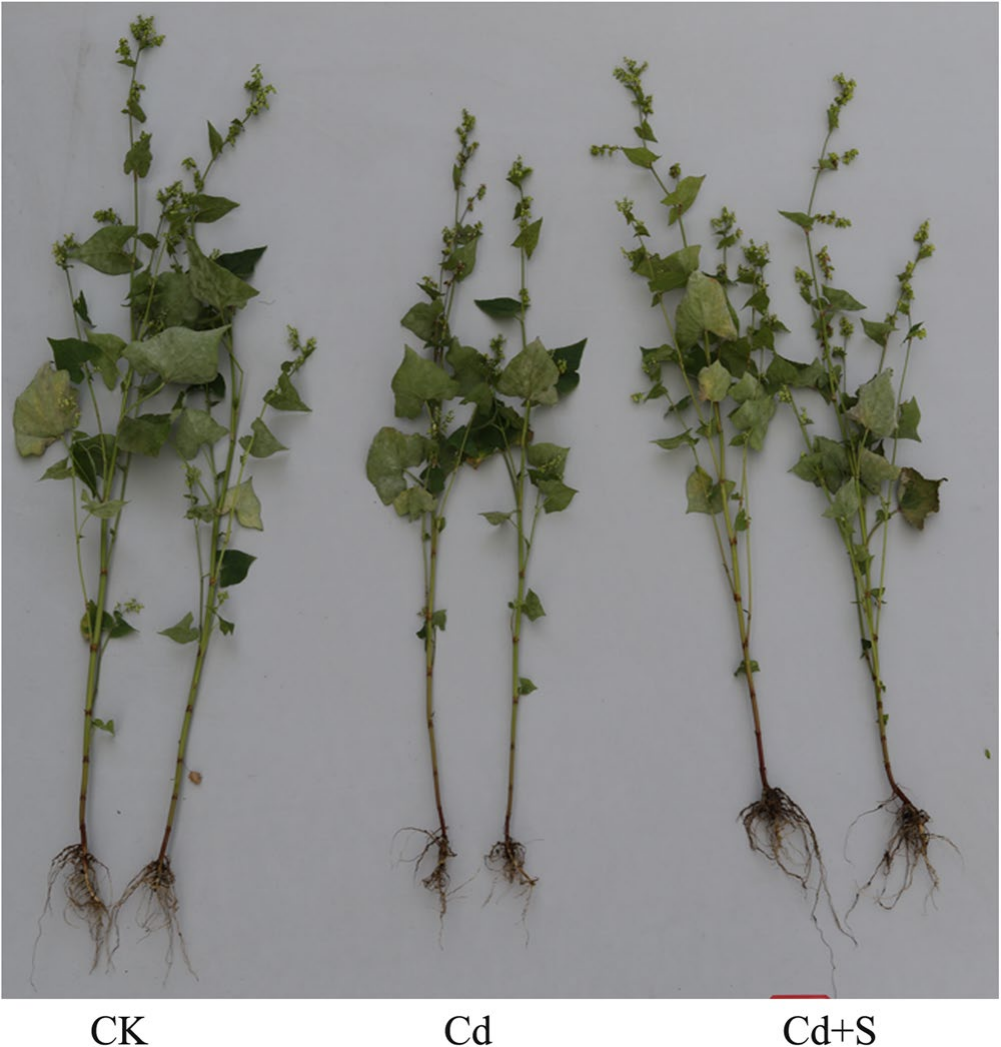
Effect of surplus S on seedling growth in the leaf and root of tartary buckwheat. Photo was taken at 10 days after treatment. CK: control plants without Cd or surplus S treatment; Cd: plants treated with cadmium; Cd + S: plants treated with cadmium and surplus sulfur.

Tartary buckwheat seedlings formed shorter roots and shoots, and a lower biomass under the Cd exposure condition. However, supplementation with S significantly increased biomass and shoot and root heights, compared with the Cd treatment alone (Fig. 1 and Table 1). Treating tartary buckwheat with Cd promoted the production of O_2_^−^ and H_2_O_2_, while net photosynthesis decreased compared to the control. However, adding 100 mM SO_4_^2−^ to plants under Cd stress increased net photosynthesis by 81.60% and decreased H_2_O_2_ and O_2_^−^ contents in leaves by 43.25% and 45.90%, respectively relative to the Cd treatment alone (Table 1). Adding SO_4_^2−^ to plant roots under Cd stress decreased H_2_O_2_ and O_2_^−^ contents by 53.48% and 51.88%, respectively compared to Cd stress alone (Table 1). These results indicate that exogenous S mitigated adverse effects on tartary buckwheat plants.

**Table 1:** Effects of surplus S on the growth, photosynthesis, and oxidative stress of Cd in leaf and root of Tartary buckwheat.

Parameters	CK	Cd	Cd + S
Shoot dry weight (g)	2.61 ± 0.13^a^	1.56 ± 0.13^c^	2.18 ± 0.15^b^
Root dry weight (g)	0.58 ± 0.06^a^	0.25 ± 0.02^b^	0.52 ± 0.03^a^
Shoot length (cm)	34.31 ± 1.64^a^	26.80 ± 1.22^b^	31.27 ± 1.38^a^
Root length (cm)	12.65 ± 0.27^a^	7.70 ± 0.16^b^	12.54 ± 0.35^c^
Net photosynthesis (µmol CO_2_⋅m^−2^⋅s^−1^)	18.75 ± 0.57^a^	6.63 ± 0.46^c^	12.04 ± 0.73^b^
Leaf O_2_^·−^ content (nmol⋅g^−1^ FW)	15.64 ± 0.73^c^	52.48 ± 0.85^a^	28.39 ± 0.94^b^
Root O_2_^·−^ content (nmol⋅g^−1^ FW)	31.08 ± 1.15^c^	86.29 ± 1.37^a^	41.52 ± 0.99^b^
Leaf H_2_O_2_ content (nmol⋅g^−1^ FW)	65.37 ± 2.25^c^	146.21 ± 2.48^a^	82.76 ± 5.74^b^
Root H_2_O_2_ content (nmol⋅g^−1^ FW)	105.63 ± 3.48^c^	254.77 ± 4.86^a^	118.51 ± 4.69^b^

The top right corner with different letters are significantly different within each group at *p* ≤ 0.05 (n = 3) according to Duncan’s multiple test. CK: control plants without Cd or surplus S treatment; Cd: plants treated with cadmium; Cd + S: plants treated with cadmium and surplus sulfur.

**Figure 2:**
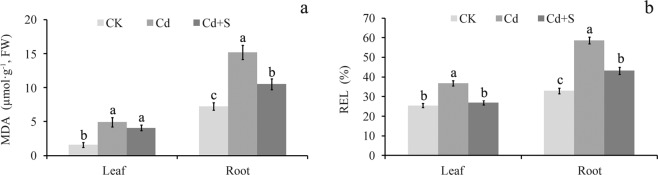
Effect of surplus S on MDA content and REL in tartary buckwheat. (**a**) Malondialdehyde (MDA) content variation in leaf and root of tartary buckwheat under Cd stress and with or without surplus S treatment. (**b**) Change of REL in leaf and root of tartary buckwheat under Cd stress and with or without surplus S treatment. Bars with different letters show the significant difference within each group at *p* < 0.05 (n = 3) according to Duncan’s multiple test. CK: control plants without Cd or surplus S treatment; Cd: plants treated with cadmium; Cd + S: plants treated with cadmium and surplus sulfur.

The MDA concentrations in leaves and roots increased significantly relative to the control after 10 days of Cd treatment (Fig. 2a). In contrast, the MDA content in roots decreased sharply under the SO_4_^2−^ treatment and Cd stress, but showed a minor decrease of MDA content in leaves (Fig. 2a). These results indicate that surplus S alleviated Cd-induced damage by minimizing MDA accumulation in the roots of tartary buckwheat.

REL was significantly enhanced in the roots and leaves of tartary buckwheat under the Cd treatment (Fig. 2b). However, the exogenous SO_4_^2−^ treatment significantly decreased REL triggered by Cd stress in leaves and roots of tartary buckwheat. This observation confirmed that applying surplus S protects against Cd-induced oxidative damage in tartary buckwheat.

### Effects of surplus S on oxidative stress

**Figure 3:**
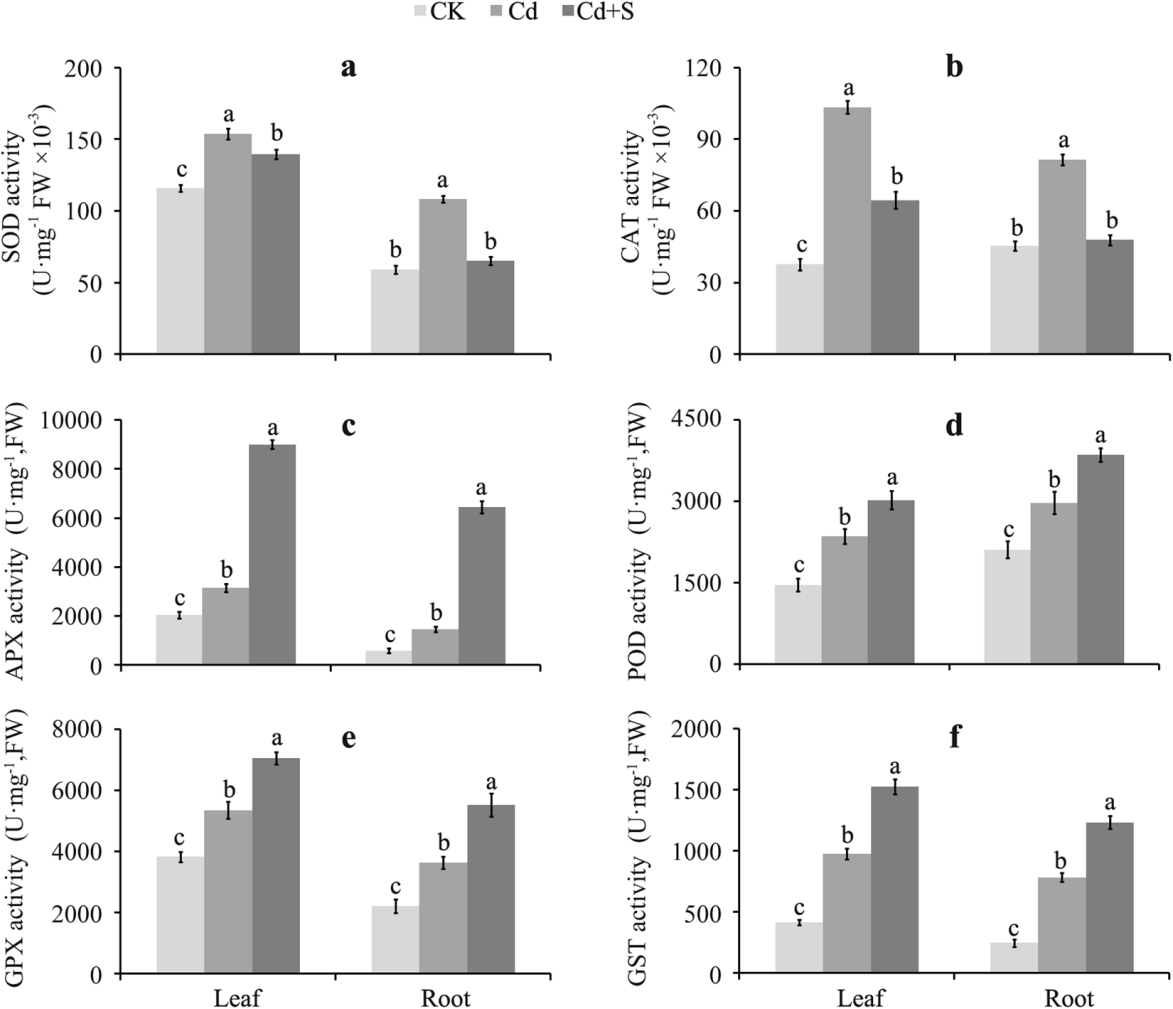
The variation of antioxidant enzyme activities in the leaf and root of tartary buckwheat exposed to Cd in the presence of surplus S. (**a**–**f**) Represent the change pattern of SOD, CAT, APX, POD, GPX, and GST activity, respectively. Bars with different letters show the significant difference within each group at *p* < 0.05 (n = 3) according to Duncan’s multiple test. CK: control plants without Cd or surplus S treatment; Cd: plants treated with cadmium; Cd + S: plants treated with cadmium and surplus sulfur.

The lipid peroxidation and electrical conductivity test results (Fig. 2 and Table 1) suggested that exogenous S treatment helps tartary buckwheat tolerate Cd stress by counteracting the accumulation of Cd-induced ROS and subsequent oxidative injury. Therefore, the ROS scavenging-associated antioxidant enzymes and/or antioxidants should be detected to verify these findings. In this study, the activities of SOD, CAT, POD, and APX, as well as the contents of AsA and GSH were investigated in tartary buckwheat. The results show that SOD, CAT, POD, and APX activities were significantly enhanced in the leaves and roots of tartary buckwheat under Cd stress compared to the control (Fig. 3a–f). However, SOD and CAT activities decreased significantly by 9.26% and 37.61% in leaves and by 39.73% and 41.32 in roots, respectively after the SO_4_^2−^ treatment, compared to the Cd treatment alone (Fig. 3a,b). APX, POD, GPX, and GST activities increased by 1.86 times, 28.64%, 31.76% and 56.05% in leaves, respectively, and increased by 3.43 times, 29.89%, 51.77% and 57.37 in roots, respectively compared to the Cd-treated plants (Fig. 3c–f).

**Figure 4:**
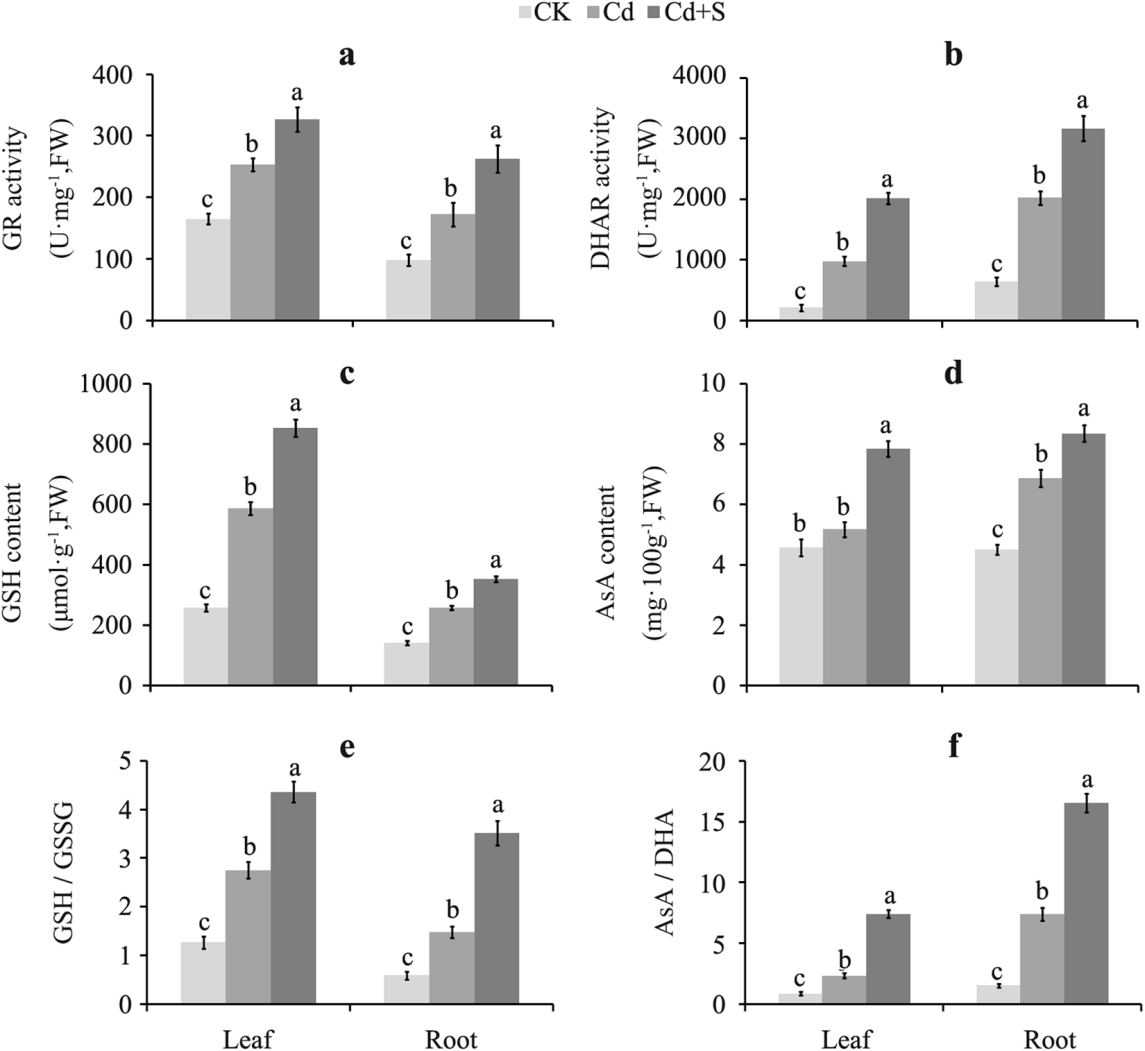
The variation of antioxidants in the leaf and root of tartary buckwheat exposed to Cd in the presence of surplus S. (**a**–**f**) Represent the change pattern of GR activity, DHAR activity, GSH content, AsA content, GSH/GSSG ratio, and AsA/DHA ratio, respectively. Bars with different letters show the significant difference within each group at *p* < 0.05 (n = 3) according to Duncan’s multiple test. CK: control plants without Cd or surplus S treatment; Cd: plants treated with cadmium; Cd + S: plants treated with cadmium and surplus sulfur.

GR and DHAR activities, which respectively play critical roles in the biosynthesis of GSH and AsA, increased by 28.67% and 106.11% in leaves and by 52.30% and 56.60% in roots after applying exogenous S under Cd stress, respectively (Fig. 4a,b). AsA and GSH contents increased consistently beyond that of only the Cd treatment, as well as the ratio of GSH/GSSG and AsA/DHA (Fig. 4c–f). These results suggest that antioxidant enzymes (SOD and CAT) played an insignificant role in surplus S-induced counteracting Cd triggered damage in tartary buckwheat, whereas the antioxidants (AsA and GSH) and antioxidant enzymes (POD, APX, GPX, and GST) had an essential function in the tolerance of Cd stress by tartary buckwheat.

### The effect of surplus S on variations in S metabolism under Cd stress

**Figure 5:**
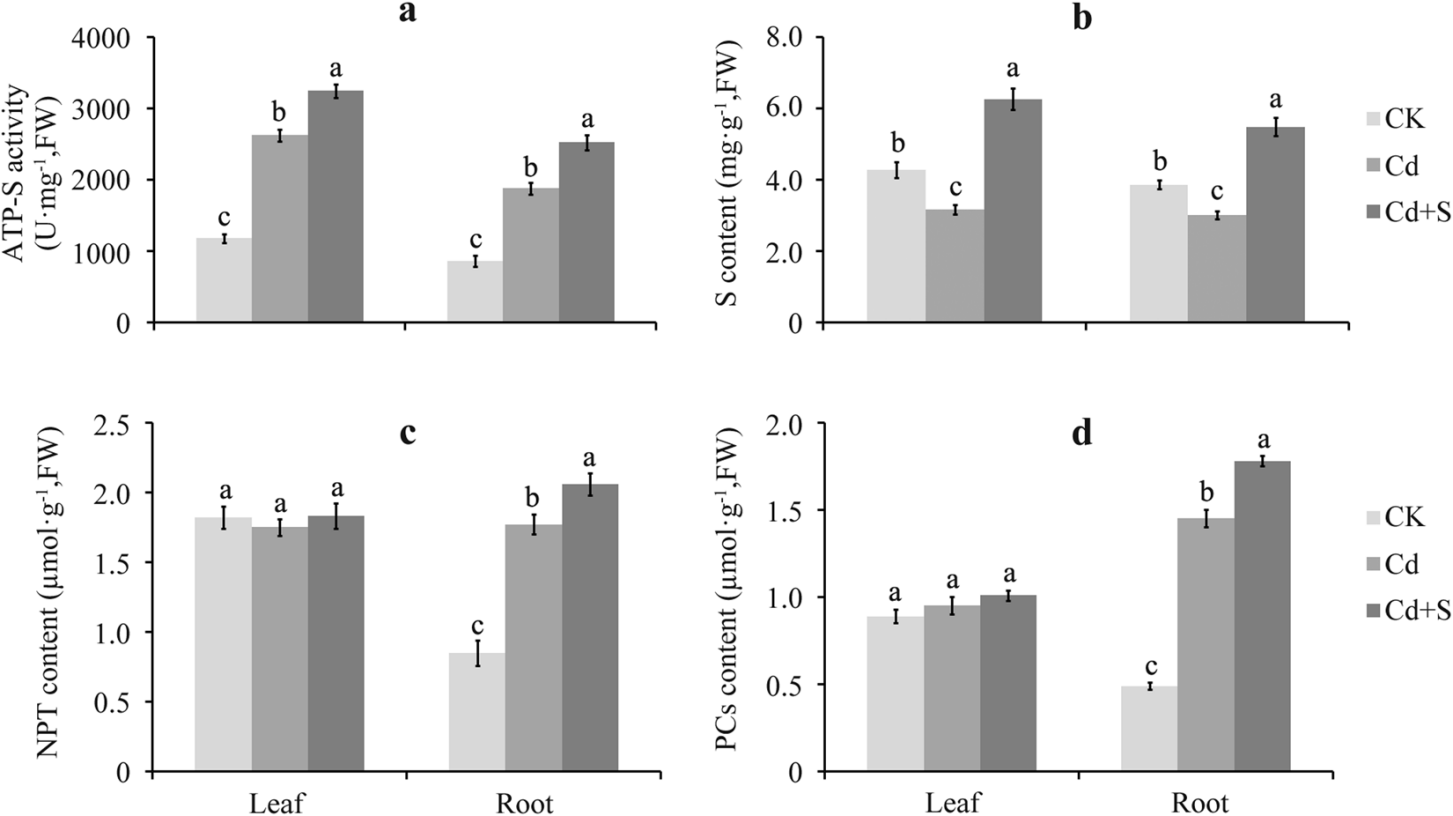
Effect of surplus S on ATP-S activity, S content, and thiol group containing peptides content in the leaf and root of tartary buckwheat. (**a**–**d**) Represent the change pattern of ATP-S activity, S content, NPT content and PCs content, respectively. Bars with different letters show the significant difference within each group at *p* < 0.05 (n = 3) according to Duncan’s multiple test. CK: control plants without Cd or surplus S treatment; Cd: plants treated with cadmium; Cd + S: plants treated with cadmium and surplus sulfur.

ATP-S activity in leaves and roots increased under the Cd treatment by 1.22 times and 1.17 times, while S content decreased by 25.76% and 21.76%, respectively compared to the control. Supplementing with S enhanced ATP-S activity by 23.70% in leaves and 34.47% in roots, and increased S content by 97.48% in leaves and 81.46% in roots compared to Cd stress alone (Fig. 5a,b).

PCs and NPT are S-containing peptides that alleviate Cd stress by chelating Cd in cells. The results show that the PC and NPT contents in leaves did not vary significantly under Cd stress, while they increased significantly in roots by 1.96 and 1.08 times, respectively compared to the control (Fig. 5c,d). Furthermore, the PC and NPT contents in the roots treated with surplus S increased by 22.76% and 16.38%, respectively compared to Cd stress only, but had little effect on leaves (Fig. 5c,d). These results suggest that Cd chelation through PCs or NPT in roots was enhanced by surplus S, while little effect was observed in leaves.

### Surplus S increases Cd sequestration in cell walls and vacuoles

The analysis of Cd concentration in different tissues of tartary buckwheat revealed a complex Cd accumulation pattern after adding surplus S. The concentration of Cd in S-treated leaves decreased by more than half compared to the Cd-treated samples. In contrast, uptake of Cd by roots increased by 90.69% (Table 2). The Cd translocation factor (Cd concentration in shoots/Cd concentration in roots) decreased from 0.29 in the Cd treatment alone to 0.06 (Table 2). A subcellular analysis of the Cd concentrations in leaves and roots was subsequently conducted. The results indicated that the Cd concentration in the cell walls of leaves increased slightly after treatment with surplus S, but did not vary significantly compared to the Cd treatment alone. The Cd accumulation in organelles and soluble fraction (containing vacuoles) parts decreased by 2.53 and 2.71 times, respectively relative to samples that were subjected to Cd stress alone (Table 3). Adding SO_4_^2−^ to roots increased Cd sequestration in the cell walls and the soluble fraction by 115.92% and 93.47%, respectively. Cd sequestration changed slightly in the organelles compared to the Cd treatment alone (Table 3). These results reveal the modulating role of exogenous S in subcellular sequestration of Cd in tartary buckwheat leaves and roots.

**Table 2:** Effects of surplus S on the distribution of Cd in leaf and root of Tartary buckwheat.

Organ	Treatment	Cd content (mg·kg^−1^, DW)
Leaf	CK	0^c^
	Cd	93.53 ± 8.37^a^
	Cd + S	36.28 ± 6.62^b^
Root	CK	0^c^
	Cd	322.13 ± 14.62^b^
	Cd + S	614.27 ± 15.53^a^

The top right corner with different letters are significantly different within each group at *p* ≤ 0.05 (n = 3) according to Duncan’s multiple test. CK: control plants without Cd or surplus S treatment; Cd: plants treated with cadmium; Cd + S: plants treated with cadmium and surplus sulfur.

**Table 3:** Subcellular analysis of Cd content under Cd and with or without surplus S.

Organ	Treatment	Cd content (mg·kg^−1^, DW)		
		Cell wall	Organelles	Soluble fraction
Leaf	CK	0^b^	0^b^	0^b^
	Cd	15.12 ± 0.75^a^	10.28 ± 0.83^a^	65.42 ± 1.54^a^
	Cd + S	14.68 ± 0.74^a^	2.91 ± 0.21^b^	17.63 ± 0.75^b^
Root	CK	0^c^	0^b^	0^c^
	Cd	38.38 ± 2.83^b^	44.36 ± 2.47^a^	236.53 ± 7.15^b^
	Cd + S	82.87 ± 2.35^a^	42.93 ± 2.98^a^	457.61 ± 8.78^a^

The top right corner with different letters are significantly different within each group at *p* ≤ 0.05 (n = 3) according to Duncan’s multiple test. CK: control plants without Cd or surplus S treatment; Cd: plants treated with cadmium; Cd + S: plants treated with cadmium and surplus sulfur.

### Effect of surplus S on *FtHMA3* and *FtHMA2* expression

**Figure 6:**
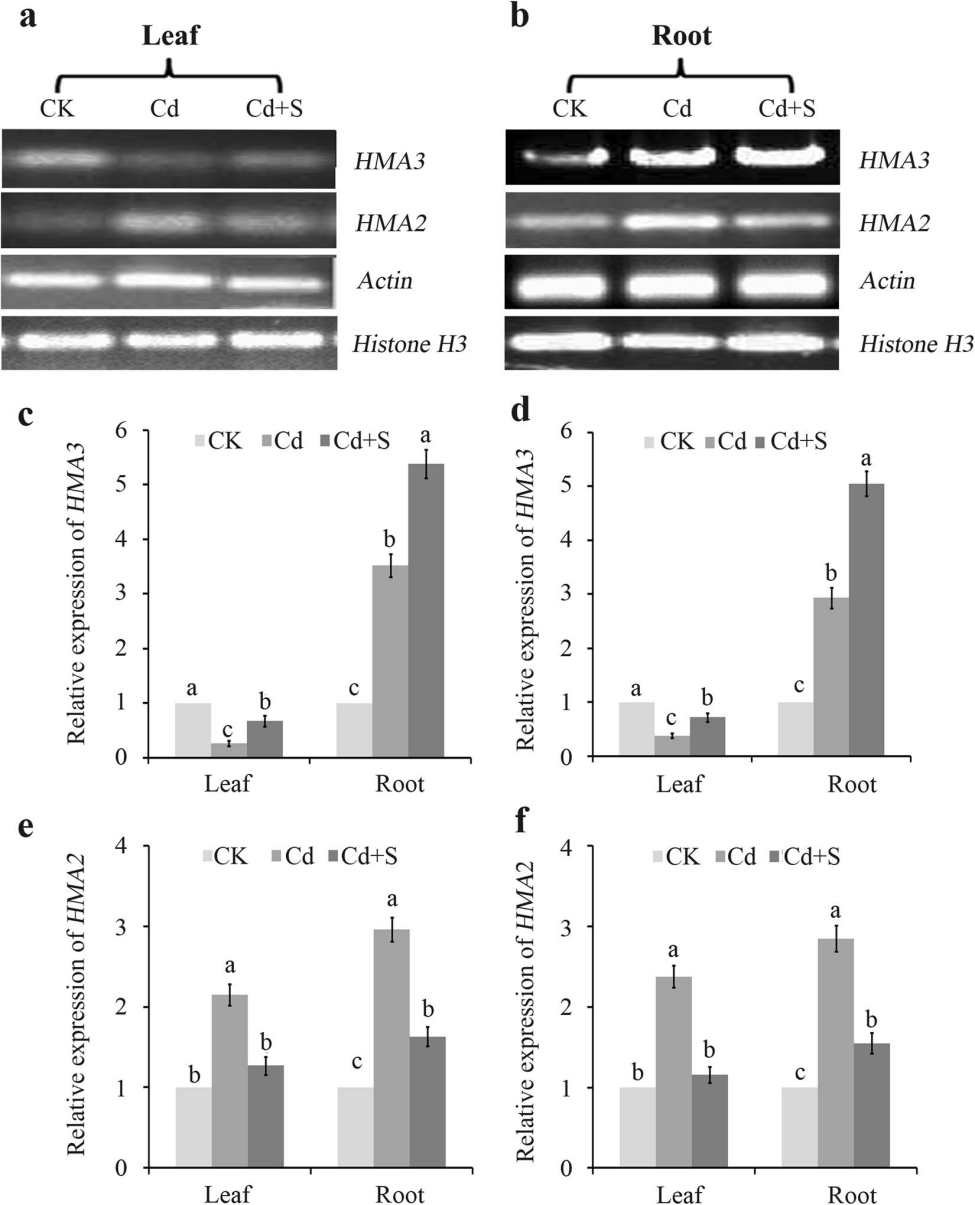
Effect of SO_4_^2−^ on expression level of *FtHMA3* in tartary buckwheat under Cd stress in the presence of surplus S. (**a**) RT-PCR analysis of *FtHMA3*; (**b**) RT-PCR analysis of *FtHMA2*; (**c**) RT-qPCR analysis of *FtHMA3* with the reference of *actin*; (**d**) RT-qPCR analysis of *FtHMA3* with the reference of *Histone H3*; (**e**) RT-qPCR analysis of *FtHMA2* with the reference of *actin*; (**f**) RT-qPCR analysis of *FtHMA2* with the reference of *Histone H3*. Bars with different letters show the significant difference within each group at *p* < 0.05 (n = 3) according to Duncan’s multiple test. CK: control plants without Cd or surplus S treatment; Cd: plants treated with cadmium; Cd + S: plants treated with cadmium and surplus sulfur.

The transcription levels of *FtHMA3* and *FtHMA2* under Cd stress and with and without surplus S were investigated. As shown in Fig. 6, *FtHMA3* mRNA levels in leaves were much lower than those in roots, indicating that *FtHMA3* is mainly expressed in roots. Further analysis showed that expression in leaves decreased significantly under Cd stress, but increased after the surplus S treatment. *FtHMA3* expression in roots was first about 2.52-fold higher in the Cd treatment alone than in control plants with reference to actin, and increased further by 52.84% in the surplus S treatment than in the Cd treatment alone (Fig. 6a,c,d). *FtHMA2* was expressed at higher levels in leaves and roots of tartary buckwheat seedlings under Cd stress compared to the control, while expression decreased significantly when additional S was supplied (Fig. 6b,e,f). These results imply that Cd translocation from roots to shoots was inhibited by the surplus S treatment, which was consistent with the variation in the Cd translocation factor (Table 2).

## Discussion

Cd is one of the most widespread pollutants in the environment, and it is toxic to plants because it induces the generation of ROS, which subsequently lead to oxidative damage, which severely inhibits plant growth^19,30^. Reports on wheat^31^ and mustard^14^ show that Cd stress results in an increase of MDA content, which is a product of lipid peroxidation and a monitor of ROS-induced oxidative stress. However, supplementary nitric oxide (NO)/S/ethylene under Cd stress can relieve Cd-induced toxicity by reducing MDA content in the roots and/or leaves of wheat and mustard^14,31^. REL is another vital index with which to evaluate the degree of oxidative stress. REL also increases under Cd stress but decreases in the roots of wheat^31^ and *Typha angustifolia*^24^ following treatment with exogenous NO. In accordance with these results, the data obtained here demonstrate that exposure to Cd significantly increased MDA content and REL, while they decreased after the surplus treatment S in both roots and leaves of tartary buckwheat, but not MDA content in leaves (Fig. 2a,b). This result suggests that exogenous S protects plant cell membranes from ROS-induced oxidative stress.

Antioxidant enzymes and non-enzymatic antioxidants have been adopted by plants to eliminate excess ROS produced under severe stress; thus, alleviating cell damage^32,33^. Here, the activities of SOD, CAT, POD, APX, GPX, and GST in leaves and roots of tartary buckwheat were all enhanced after Cd exposure, demonstrating that Cd induced oxidative stress and further indicates that tartary buckwheat is capable of tolerating Cd by increasing the activities of antioxidant enzymes (Fig. 3a–f). In contrast, the surplus S treatment decreased SOD and CAT activities, compared to samples that were exposed to Cd alone. These results are consistent with the findings of Wu *et al*. (2015), who showed that supplying S to rice seedlings decreases Cd-induced increases of SOD and CAT activities^34^. However, it has been reported that supplying S enhances Cd tolerance in *Panicum maximum* cv. Tanzania by increasing amino acid synthesis and antioxidant enzyme activities^35^. This contradictory result suggests that a different mechanism is applicable in different plant species. Coincidently, the activities of APX, POD, GPX, and GST further improved after treatment with exogenous S compared to Cd stress alone (Fig. 3c–f). These results might be attributed to the capability of surplus S to scavenge Cd-triggered ROS through other antioxidant enzymes (including APX, POD, GPX, and GST).

AsA and GSH as the most abundant and important non-enzyme antioxidants, and they increased significantly in response to the surplus S treatment of Cd exposed plants, compared to those exposed to Cd alone (Fig. 4c,d). GR is the rate-limiting enzyme in the AsA-GSH cycle, which catalyzes the reaction of reducing GSSG (oxidative state of GSH) to GSH with the electron donor NADPH^17,18^. DHAR is a critical enzyme responsible for the biosynthesis of AsA by reducing DHA (oxidative state of AsA) to AsA^17,18^. GR and DHAR activities both increased in response to Cd stress alone, and increased further after adding S (Fig. 4a,b). Consistent with the change in the GR and DHAR activity pattern, the ratio of GSH/GSSG and AsA/DHA also increased under the Cd stress alone and excess S under Cd stress conditions (Fig. 4e,f). These results indicate that supplying additional S alleviated Cd toxicity by enhancing the AsA-GSH cycle, which is in accordance with the findings of previous reports^17,18,20^.

An alternative mechanism to embed heavy metal chelates in plant cells via high-affinity groups is adopted by plants to detoxify heavy metals. Several thiol-containing groups, including NPT, PCs, and cysteine are critical high-affinity groups that play a vital role in the detoxification and homeostasis of heavy metals in plants^36,37^. Particularly PCs, which chelate Cd in the cytosol as a low molecular weight complex that is transported into the vacuole where they ultimately form a more stable storage state of high molecular weight Cd and PC complexes^21^. GSH, NPT, and PC biosynthesis is dependent on S assimilation. ATP-S catalyzes the reaction between ATP and sulfate to form adenosine phosphosulfate, which is the initial step in the S assimilation pathway. ATP-S activity increases with Cd stress, and increases further when excess S is supplied^14,17,18,20,21^. In the present study, ATP-S activity increased in the leaves and roots of tartary buckwheat under Cd stress, and increased further when exogenous S was added compared to the Cd treatment alone (Fig. 5a). However, total S contents in leaves and roots of tartary buckwheat first decreased under Cd stress and then increased after supplementation with S (Fig. 5b), suggesting that the Cd–S interaction improved sulfate assimilation in both leaves and roots of tartary buckwheat, thereby satisfying the increased demand for GSH, NPT, or PC synthesis under Cd stress^18^.

These results show that GSH content improved under the Cd and surplus S treatments. However, some differences in NPT and PC contents were evident between roots and leaves of tartary buckwheat under the Cd and surplus S treatments. NPT and PC contents increased significantly in roots upon exposure to Cd. Supplementation with S further increased both NPT and PC contents in roots compared to the Cd exposure alone treatment. In contrast, NPT and PC contents did not change significantly in leaves among any of the treatments (Fig. 5c,d). These results suggest that PCs and NPT play an important role in Cd sequestration into the vacuole after surplus S and Cd are added to the roots of tartary buckwheat, while other mechanisms may play an important role detoxifying Cd in leaves, including the aforementioned antioxidant enzymes (APX, POD, GPX, and GST) and antioxidant agents (AsA and GSH).

To verify the speculation that surplus S aids Cd sequestration into the vacuole, the Cd concentrations in leaves and roots of tartary buckwheat were further analyzed in response to Cd stress. The results illustrated that Cd accumulation in leaves was clearly inhibited by the surplus S treatment, while the Cd concentration in roots increased significantly, indicating a reduced Cd translocation factor (Table 2). A subcellular distribution analysis showed that the concentration of Cd in the vacuole-containing fraction (i.e., soluble fraction) of roots increased significantly while it decreased in leaves (Table 3). Nevertheless, NPT and PC contents tended to change in leaves, the Cd concentration in leaves decreased significantly, and the tendency to decrease accounted for the decreased root-to-shoot Cd translocation factor and expression of *FtHMA2*, a plasma membrane Cd extruder that aids in Cd translocation from roots to shoots. This result was consistent with the finding of Hu *et al*.^38^ that silencing *SaHMA2* results in increased Cd accumulation in roots but a decrease in leaves. The processes that control Cd translocation from roots to shoots are much more associated with roots than shoots. In this case, Cd translocation in plants supplied with S decreased because the synthesis of NPT and PCs increased in roots, and Cd was bound to the root cell wall and was sequestered in the vacuoles, which is consistent with the findings in mustard and *Typha angustifolia* under Cd and exogenous NO treatments reported by Per *et al*.^19^ and Hui *et al*.^24^, respectively. They conclude that NO improves S assimilation and GSH production to decrease root-to-shoot translocation of Cd; thus, further alleviating Cd toxicity in plants^19–21^. Liang *et al*. (2016) revealed an ameliorative role of S in protecting *Brassica chinensis* L from Cd toxicity by enhancing the AsA-GSH cycle and PCs synthesis, and inhibiting Cd translocation from roots to shoots^18^. Our observations were in accordance with these findings. Interestingly, the expression of *FtHMA3*, a cadmium transporter that plays a critical role directly transporting Cd from the cytosol into the vacuole^39^, was mainly expressed in tartary buckwheat roots and significantly increased with surplus S under the Cd stress treatment (Fig. 6a–c). This is important evidence demonstrating that surplus S improved Cd translocation in the vacuoles of tartary buckwheat roots, and reduced Cd content in leaves. Cao *et al*. (2018) also reported that excessive S supply results in overexpression of *OsHMA3* in rice roots, and reduces Cd accumulation in rice shoots and grains, but increases Cd content in roots^21^. Nonetheless, the influence of supplementary S on the expression of *OsHMA3* and its relationship to Cd translocation needs to be further investigated.

Cd accumulation was also enhanced in the root cell walls of tartary buckwheat (Table 3). It is well known that the cell wall is key component to relieve Cd toxicity in plants. Previous reports have shown that supplying NO alleviates Cd stress by increasing the activity of proteins, such as oxalate oxidase, as well as pectin and hemicellulose contents in root cell walls that can bind heavy metals and subsequently increase Cd content in roots, while decreasing Cd accumulation in leaves^40,41^. Supplying adequate S to Massai grass exposed to Cd increases root length, the root surface, and increases Cd content of the cell walls by depositing suberin and lignin in the endodermis and during development of the G-layer^42^. Supplying S reduces Cd uptake and translocation in rice shoots and grains by improving iron plaque formation on the rice root surfaces^21^. These results suggest that surplus S could lessen Cd toxicity in tartary buckwheat by enhancing the Cd holding functions of root cell walls; thus, balancing various physiological and biochemical functions in the cytoplasm under a Cd environment. There may be an S-dependent mechanism to exclude Cd from the cell and reduce toxic Cd accumulation in the cytoplasm in tartary buckwheat; however, this model requires further investigation before it can be verified.

## Conclusion

Adding the appropriate amount of S tended to increase plant height, root length, and root and shoot dry weights of tartary buckwheat seedlings, and significantly reduced Cd accumulation in leaves of tartary buckwheat in the absence or presence of Cd. A further analysis indicated that surplus S mitigated Cd stress in tartary buckwheat mainly by enhancing the role of cell wall Cd binding, Cd chelation, and vacuolar sequestration in tartary buckwheat roots by improving PC and NPT biosynthesis, increasing *FtHMA3* expression, and decreasing *FtHMA2* expression. Consequently, root-to-shoot translocation of Cd was inhibited, and the Cd content in leaves of tartary buckwheat decreased. This study also showed that the enhanced effect of surplus S on antioxidant agents (AsA and GSH) and antioxidant enzymes (APX, POD, GPX, and GST) may play an important role balancing the ROS level in leaves and roots of tartary buckwheat, while the antioxidant enzymes (SOD and CAT) exerted little effect on scavenging additional ROS.
